# The distinct fate of smooth and rough *Mycobacterium abscessus* variants inside macrophages

**DOI:** 10.1098/rsob.160185

**Published:** 2016-11-30

**Authors:** Anne-Laure Roux, Albertus Viljoen, Aïcha Bah, Roxane Simeone, Audrey Bernut, Laura Laencina, Therese Deramaudt, Martin Rottman, Jean-Louis Gaillard, Laleh Majlessi, Roland Brosch, Fabienne Girard-Misguich, Isabelle Vergne, Chantal de Chastellier, Laurent Kremer, Jean-Louis Herrmann

**Affiliations:** 1 UMR1173, Inserm and UFR Des Sciences de la Santé Simone Veil, Université de Versailles Saint Quentin, Montigny, France; 2 UMR1179, Inserm and UFR Des Sciences de la Santé Simone Veil, Université de Versailles Saint Quentin, Montigny, France; 3 Centre National de la Recherche Scientifique FRE 3689, Centre d’études d'agents Pathogènes et Biotechnologies pour la Santé, Université de Montpellier, 1919, Route de Mende, 34293, Montpellier, France; 4 Centre d'Immunologie de Marseille-Luminy, Aix-Marseille Université UM 2, Inserm, U1104, CNRS UMR7280, 13288, Marseille, France; 5 CNRS, Institut de Pharmacologie et de Biologie Structurale (IPBS), UMR 5089 CNRS/Université Paul Sabatier, 205 route de Narbonne, BP 64182, 31077 Toulouse Cedex 4, France; 6 Unité de Pathogénomique mycobactérienne, Institut Pasteur, 25 rue du Dr Roux, 75724 Paris Cedex 15, Paris, France; 7 INSERM, CPBS, 34293 Montpellier, France

**Keywords:** *Mycobacterium abscessus*, macrophages, phagosome, innate response, rapid-growing mycobacteria

## Abstract

*Mycobacterium abscessus* is a pathogenic, rapidly growing mycobacterium responsible for pulmonary and cutaneous infections in immunocompetent patients and in patients with Mendelian disorders, such as cystic fibrosis (CF). *Mycobacterium abscessus* is known to transition from a smooth (S) morphotype with cell surface-associated glycopeptidolipids (GPL) to a rough (R) morphotype lacking GPL. Herein, we show that *M. abscessus* S and R variants are able to grow inside macrophages and are present in morphologically distinct phagosomes. The S forms are found mostly as single bacteria within phagosomes characterized by a tightly apposed phagosomal membrane and the presence of an electron translucent zone (ETZ) surrounding the bacilli. By contrast, infection with the R form leads to phagosomes often containing more than two bacilli, surrounded by a loose phagosomal membrane and lacking the ETZ. In contrast to the R variant, the S variant is capable of restricting intraphagosomal acidification and induces less apoptosis and autophagy. Importantly, the membrane of phagosomes enclosing the S forms showed signs of alteration, such as breaks or partial degradation. Although not frequently encountered, these events suggest that the S form is capable of provoking phagosome–cytosol communication. In conclusion, *M. abscessus* S exhibits traits inside macrophages that are reminiscent of slow-growing mycobacterial species.

## 1. Introduction

The *Mycobacterium* genus represents a complex group of more than 100 species, of which only a limited number are strict human or animal pathogens. Most of the non-pathogenic saprophytic mycobacteria belong to the rapid-growing mycobacteria (RGM) group, although some of them, including *M. abscessus*, *M. chelonae* and *M. fortuitum*, have recently been classified as true opportunistic pathogens [1]. *Mycobacterium abscessus* is now recognized as the major pulmonary pathogen within the RGM [2], with cystic fibrosis (CF) patients being particularly vulnerable to infection with this mycobacterium [3–7]. *Mycobacterium abscessus* is also regarded as a nosocomial infectious agent responsible for several epidemics due to clinical practices with contaminated materials [8,9]. Recent epidemiological studies and clinical case studies of CF patients infected with *M. abscessus* emphasized the persistence, sometimes for several decades, of *M. abscessus* within the host [10–12]. Finally, *M. abscessus* has been associated with the most direct impact on lung functions in CF patients when compared with the slow-growing mycobacterium (SGM) *M. avium*, and non-fermentative Gram-negative bacteria [13].

Like *M. avium* or *M. smegmatis* [14], *M. abscessus* displays two distinct morphotypes on solid agar media: a smooth (S) variant, non-cording but motile and biofilm-forming, and a rough (R) variant, cording but non-motile and non-biofilm-forming. The major difference between these two variants resides in the total loss of surface-associated glycopeptidolipids (GPL) in the R form [15]. Importantly, the R variant appears to arise only during the course of infection in the host organism, as evidenced by culture-positive sputum samples from patients [11] or experiments in B-cell-deficient mice [16]. In addition, R variants are frequently associated with severe infections as observed in CF patients infected with *M*. *abscessus* [11,12]. In the light of these findings, one may hypothesize that S and R variants differentially affect the phagocytic pathway.

One key difference between pathogenic and non-pathogenic mycobacteria is the capacity of pathogenic mycobacteria to survive and replicate within macrophages (Mϕ) and dendritic cells (DC) by arresting phagosome maturation and, hence, preventing fusion with lysosomes [17–23]. *Mycobacterium fortuitum* and *M. smegmatis*, a saprophytic RGM, are both unable to multiply inside Mϕ and are rapidly cleared from the infected cells [24,25]. In sharp contrast, *M. abscessus* not only survives, but also replicates inside Mϕ [26–28]. Histopathological studies performed on autopsy-derived lung tissue sections of patients who died from an infection with *M*. *abscessus* revealed the presence of granulomas with caseous lesions, a hallmark of persistent mycobacterial infection [29]. Such characteristic features have also recently been corroborated in zebrafish and mice infected with *M. abscessus* [16,30].

Based on these physiopathological features, *M. abscessus* can be regarded as a pseudotuberculous and virulent RGM with a potential dual pathogenic manifestation linked to the S to R transition. This prompted us to compare the phagocytic uptake and fate of both S and R variants within Mϕ with respect to growth and describe how these events affect the endomembrane compartment in which they reside. Our findings point to intracellular characteristics that are specific to *M. abscessus* S and which resemble those usually attributed to pathogenic SGM.

## 2. Results

### 2.1. Differential phagocytic uptake of *Mycobacterium abscessus* S and R variants

**Figure 1.**
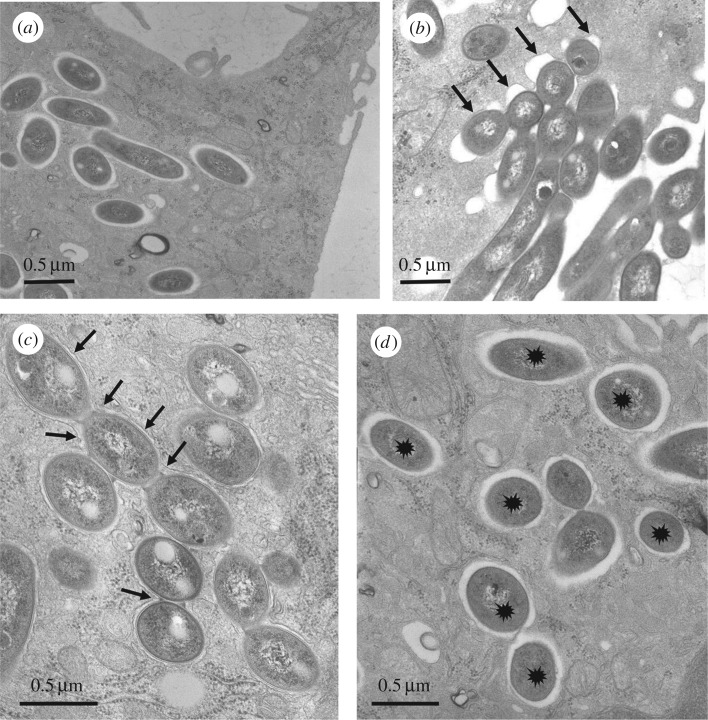
Ultrastructural differences of the *M. abscessus* S- or R-containing phagosomes. Bone marrow-derived murine Mϕ (BMDM) were infected with either the S (*a*,*d*) or R (*b*,*c*) variants of *M. abscessus* for 3 h. At 24 h p.i., cells were fixed and processed for TEM. Thin sections were analysed for phagocytic uptake of each variant (*a*,*b*) and the morphological appearance of R (*c*) or S (*d*)-containing phagosomes. S forms (*a*) reside within phagosomes and none are found in phagocytic cups whereas most of the R forms (*b*) are still clustered in phagocytic cups (arrows) at 24 h p.i. (*c*) Once formed, the R-containing phagosomes usually comprise several bacteria (social phagosomes, indicated with arrows). (*d*) The S-containing phagosomes typically comprise a single bacterium (loner phagosomes, indicated with stars).

Bone-marrow-derived murine Mϕ (BMDM) were infected with the S or R variant of *M. abscessus* at a multiplicity of infection (MOI) of 1 in the absence of antibiotics (see Experimental procedures). After extensive washes to eliminate the residual extracellular bacteria, cells were fixed and processed for transmission electron microscopy (TEM) at selected time points thereafter (0–24 h p.i.). Examination of thin sections up to 24 h p.i. showed that the S variant was efficiently phagocytized. Bacteria in phagocytic cups or still binding to the cell surface were usually not found with *M. abscessus* S (figure 1*a*). Around 80% of the phagosomes harbouring *M. abscessus* S were loner phagosomes containing a single bacterium whereas 60% of the phagosomes harbouring *M. abscessus* R were social phagosomes with at least 2 bacilli (electronic supplementary material, figure S1). Most of the cell profiles displayed between 1 and 10 phagosomes at 24 h p.i. By contrast, the cell profiles displayed a low number of phagosomes following infection with the R variant. This could be linked to the highly aggregative nature of the R variant, despite extensive treatments involving several passages through a syringe needle. An important consequence of the R clumping/cording is that, in many instances, bacteria were gathered in long chains located at the close vicinity of the cell surface or in phagocytic cups. In the latter case, the tips of the pseudopods seemed to be unable to fuse together to give rise to nascent phagosomes (figure 1*b*). This observation indicates that a large amount of R forms might remain outside the cells. A few phagosomes, however, were found within Mϕ. They usually harboured large numbers of the *M. abscessus* R variant (figure 1*c*) as opposed to those harbouring the S variant, usually containing a single bacterium (figure 1*d*).

### 2.2. *Mycobacterium abscessus* multiplies similarly inside wild-type and cystic fibrosis conductance transmembrane regulator defective Mϕ

**Figure 2.**
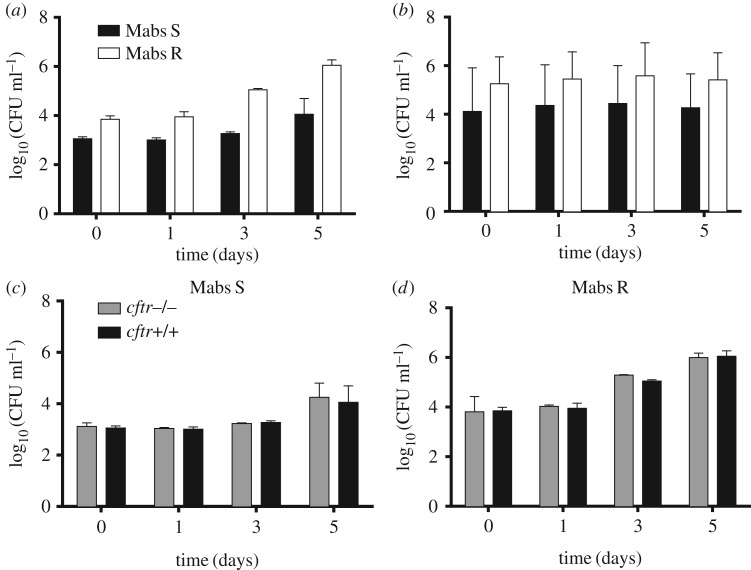
Growth of *M. abscessus* S (Mabs S) and R (Mabs R) variants in different cells. Wild-type murine (*a*) or human (*b*) Mϕ were infected with S and R variants. Amikacin treatment was applied to avoid extracellular growth (see Experimental procedures). The number of CFU was determined at the indicated times p.i. (*c*,*d*) Wild-type and *cftr*^−/−^ murine Mϕ were infected as mentioned above. Intracellular growth was similar in murine wild-type Mϕ and in murine Mϕ knock-out for *cftr* (*cftr*^−/−)^. Error bars indicate the s.e.m., based on the results from three independent experiments.

We next evaluated the intracellular growth of both variants in murine and human Mϕ by infecting cells at an MOI of 1 for 3 h (see Experimental procedures). That *M. abscessus* S and R survive in murine (figure 2*a*) and human (figure 2*b*) Mϕ is in line with previous reports [26,28]. However, it was not possible to directly compare the data obtained for the S and R variants because we systematically observed important differences in the mycobacterial uptake (up to a one log_10_ difference for the R variant). Determination of bacterial doubling time in Mϕ was 14.3 ± 0.8 h for the R variant and 19.4 ± 0.6 h for the S variant (figure 2*a,b*) compared with 6.0 ± 0.6 h and 5.8 ± 0.7 h for the *in vitro* grown R and S variants, respectively (electronic supplementary material, figure S2). These differences might be due to the intrinsic clumping of the *M. abscessus* R variant (figure 1*b*,*c*), despite extensive treatments to obtain homogeneous bacterial preparations [21,31]. Although extracellular growth cannot be totally excluded, this seems very unlikely because cells were systematically maintained under amikacin treatment during the duration of the experiment.

*Mycobacterium abscessus* infection has been reported in patients with CF, a genetic disease linked to a functional defect of the CFTR (cystic fibrosis conductance transmembrane regulator) chloride channel [32]. We therefore evaluated whether mutations abolishing the expression level or function and/or the stability of CFTR may affect *M. abscessus* intracellular survival/growth. Comparison of the bacterial loads clearly indicated that intracellular growth of the S and R variants was similar in wild-type and *cftr*^−/−^ BMDM (figure 2*c*,*d*). Comparable results were obtained following infection of murine Mϕ carrying the CFTR^ΔF508^ mutation, representing the most frequent mutation encountered in CF patients and resulting in an abnormal CFTR protein (electronic supplementary material, figure S3). Overall, these observations not only indicate that *M. abscessus* can resist the bactericidal activity of murine/human Mϕ but also suggest that a functional CFTR is not required for sustaining intracellular growth of *M. abscessus*, at least within *in vitro* infected Mϕ.

### 2.3. Distinct morphology of the intraphagosomal *Mycobacterium abscessus* S and R variants

**Figure 3.**
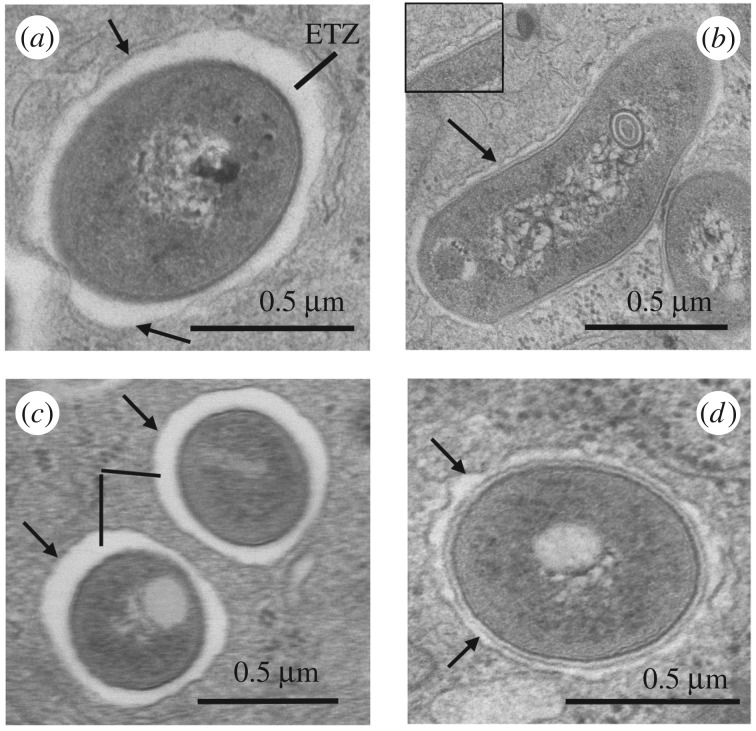
Morphological appearance of the electron translucent layer (ETZ) of the cell wall of *M. abscessus* within phagosomes. Bone marrow-derived murine Mϕ (BMDM) were infected with various *M. abscessus* strains for 3 h. At selected time points p.i., cells were fixed and processed for TEM. The cell wall ultrastructure of the different strains was assessed on 100–200 different bacterial profiles. (*a*) S variant: the electron translucent outermost layer (ETZ) of the wall was thick and apposed to the smooth phagosome membrane (arrows) all around. (*b*) R variant: in the absence of GPL production, the ETZ was thin and the phagosome membrane had a wavy appearance (arrows); in some instances a close contact was observed at discrete sites (arrow and insert). (*c*) *Mycobacterium abscessus* Δ*mmpL4b* complemented strain: as for the S variant. (*d*) *Mycobacterium abscessus* Δ*mmpL4b* mutant: no GPL produced and thin ETZ as for the R variant.

The persistence phenotype of *M. abscessus* S and R in Mϕ prompted us to examine their ultrastructure within phagosomes. The most prominent feature was the appearance of the mycobacterial cell wall and its interaction with the phagosome membrane. In the case of the S strain, the outermost electron translucent zone (ETZ), which is a major part of the mycobacterial cell wall [33], was thick and apposed to the phagosome membrane all around (figure 3*a*). By contrast, the R form displayed a very thin ETZ (figure 3*b*). Although the phagosome membrane was no longer tightly apposed to the mycobacterial cell wall all around, it did seem to make contact with the mycobacterial cell wall at discrete sites (figure 3*b*, arrows and insert).

It is well known that a major difference in the cell wall of the S and R variants of *M. abscessus* and *M. bolettii* resides in the lack of GPLs in the outermost cell wall layer of the R variant [34,35]. To confirm the dependence of ETZ formation on GPL production, BMDM were infected with a Δ*mmpL4b* mutant generated in an S background, a well-defined mutant that fails to produce and export GPL but exhibits a clumping/cording phenotype similar to the wild-type R strain [34]. BMDM were also infected with the corresponding complemented strain expressing *mmpL4b* under the control of the *hsp60* promoter on the pNVB1 integrative plasmid [36]. Only the wild-type *M. abscessus* S and the Δ*mmpL4b* complemented strain, both producing GPLs, elaborated a thick ETZ (figure 3*a*,*c*), whereas the GPL-deficient Δ*mmpL4b* mutant, like the R variant, did not (figure 3*b*,*d*). Overall, these results indicate that *M. abscessus* elaborates an ETZ that depends on GPL production, which might contribute to the survival of the S variant within its host cell.

### 2.4. *Mycobacterium abscessus* S prevents phagosome maturation/acidification

**Figure 4.**
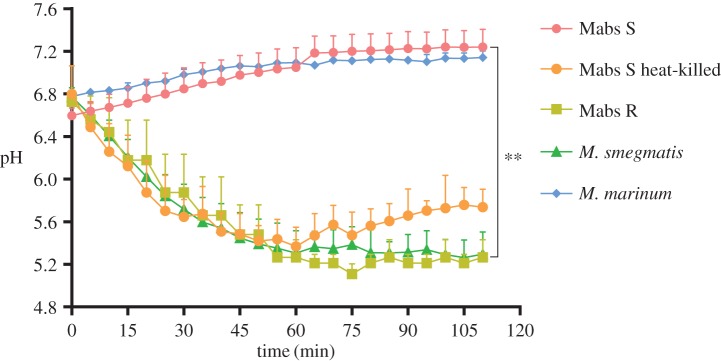
Absence of phagosomal acidification inside *M. abscessus* S-infected Mϕ. Human THP-1 Mϕ were infected with FITC-labelled mCherry-expressing *M. abscessus* S (Mabs S) and R (Mabs R), *M. smegmatis*, *M. marinum* and heat-killed *M. abscessus* S (Mabs S heat-killed). Fluorescent signals were measured sequentially at 485 nm (FITC) and 544 nm excitation wavelengths (mCherry) after a 15 mn incubation period at 37°C. The first time point on the *x*-axis (0 mn) was taken immediately after the 15 mn incubation. Subsequently, the pH at each time point was extrapolated from a standardized pH curve. The pH values were significantly different depending on whether phagosomes contained S or R variants. Results are representative of three independent experiments. (**p < 0.01).

Based on the intracellular survival of the two variants, it was of importance to understand by which mechanism(s) they resist the bactericidal response, known to clear most RGM infections. Live pathogenic mycobacteria, but not avirulent mycobacteria, are known to be retained in a mildly acidified environment [17,37–39]. The measurement of the phagosomal pH, following internalization, has been widely used as a physiologically relevant indicator of the maturation status of phagosomes [40]. We therefore analysed the pH of S- versus R-containing phagosomes in Mϕ using direct measurement of intraphagosomal pH by double fluorescent labelling. FITC (pH sensitive)-labelled *M. abscessus* S or R expressing mCherry (pH resistant) were used to infect Mϕ. The ratio between pH-sensitive and pH-resistant fluorescent stains was scored at specific time points. The pH was then calculated by comparing each ratio against a pH standard curve established with well-defined pH buffers [40]. *Mycobacterium abscessus* S- and *M. marinum*-containing phagosomes were not acidified in murine Mϕ, as reported for *M. tuberculosis* or *M. avium* infected cells [17,37] (figure 4). That heat-killed *M. abscessus* S failed to prevent phagosomal acidification suggests that this relies on an active process. Conversely, *M. abscessus* R-containing phagosomes were significantly more acidic than those containing the S variant at all time points (figure 4). A fast decline in phagosomal pH was previously reported to correlate with the rapid processing of phagosomes in late phagosomes or phagolysosomes [41]. This establishes that *M. abscessus* S-containing phagosomes are not processed into phagolysosomes following infection as shown earlier for phagosomes containing pathogenic mycobacteria [17,23,42–44].

### 2.5. The S variant may induce phagosome-cytosol communication

**Figure 5.**
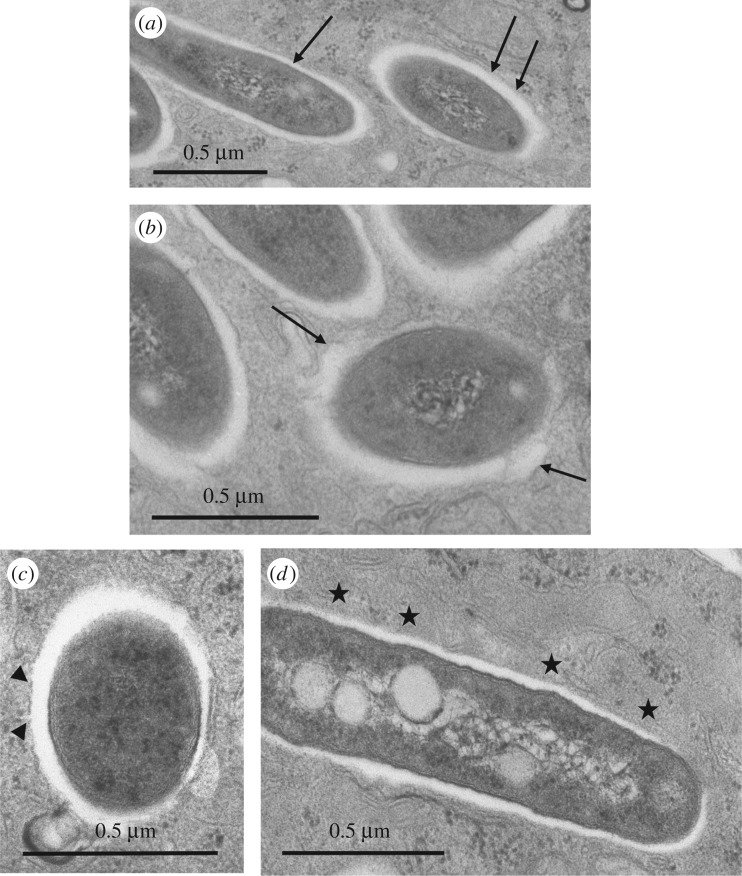
Alteration of the membrane of phagosomes containing the S variant as assessed by TEM. Bone marrow-derived murine Mϕ were infected with *M. abscessus* S for 3 h. Phagosomes were examined for obvious signs of membrane alteration/destruction. (*a*) No alteration: the phagosome membrane is smooth (arrows) and closely apposed to the mycobacterial cell wall ETZ all around. (*b*) First sign of alteration: the phagosome membrane has become wavy and is no longer closely apposed to the bacterium all around (arrows). (*c*) The phagosome membrane displays breaks (arrowheads). (*d*) The phagosome membrane is no longer visible (stars).

Both *in vitro* and *in vivo* studies demonstrated that pathogenic *M. tuberculosis* and *M. marinum* induce ruptures in the phagosome membrane and establish a phagosome-to-cytosol communication prior to the host cell death [45–48]. We evaluated whether this may also occur for *M. abscessus.* Conventional TEM approaches were first used to investigate whether the S form was able to promote an alteration of the phagosomal membrane**,** eventually followed by rupture of the membrane, which would allow the S form to gain access to the cytosol. Phagosomal rupture and phagosome-cytosol communication can be difficult to assess by conventional TEM because of tangential sections through part or all of the phagosome membrane. Care was therefore taken to examine only parts of the phagosome membrane where the plane of the section was perpendicular to the mycobacterial cell wall and hence also the phagosome membrane. Four situations were observed at 24 h p.i., as follows. (i) In the first and most frequent case, the phagosome membrane had a smooth appearance and it was tightly apposed to the ETZ (figure 5*a*, arrows). (ii) For part of the phagosomes, the membrane became wavy and it was no longer closely apposed to the ETZ (figure 5*b*, arrow). This could represent the first sign of an alteration of the phagosomal membrane. (iii) In rare instances, interruptions in the phagosome membrane were observed (figure 5*c*, arrowheads) that were not observed in other endocytic organelles (not shown). (iv) Finally, in a few cases, the phagosome membrane was no longer visible around part of the bacterium (figure 5*d*). Owing to the above-mentioned constraints, it was not possible by TEM to determine whether the entire bacterium had been freed in the cytosol (complete rupture of the phagosome membrane) or not (phagosome–cytosol communication only) and quantifications were not possible for the same reasons.

Phagosome membrane rupture was subsequently assessed using the CCF-4 (cephalosporin core linking 7-hydroxycoumarin to fluorescein) substrate in a fluorescence resonance energy transfer (FRET) assay, as previously described [47]. Differentiated THP-1 cells infected at an MOI of 1, either with *M. abscessus* S, *M. smegmatis* (negative control) or *M. marinum*, displayed at day 0 (3 h p.i.) a FRET 450 nm (blue)/535 nm (green) ratio of 1 (electronic supplementary material, figure S4), indicative of an absence of CCF-4 cleavage by the *M. abscessus* β-lactamase (Bla_Mab_) [48]. Interestingly, and in contrast to *M. smegmatis*-infected Mϕ, *M. abscessus* S-infected cells displayed a FRET ratio of 1.5 at 24 h p.i., indicative of the fluorescence shift as a result of the disruption of CCF-4 by Bla_Mab_ in the cytosolic compartment (electronic supplementary material, figure S4). The FRET assay, performed in THP-1 cells, relies either on phagosome–cytosol interplay enabling diffusion of Bla_Mab_ into the cytosol and/or on the presence of free intra-cytosolic organisms. These results corroborate the TEM observations in murine Mϕ and suggest that the S variant, but not the R form, might establish a successful phagosome–cytosol communication.

### 2.6. *Mycobacterium abscessus* S fails to trigger apoptosis and bacterial autophagy

**Figure 6.**
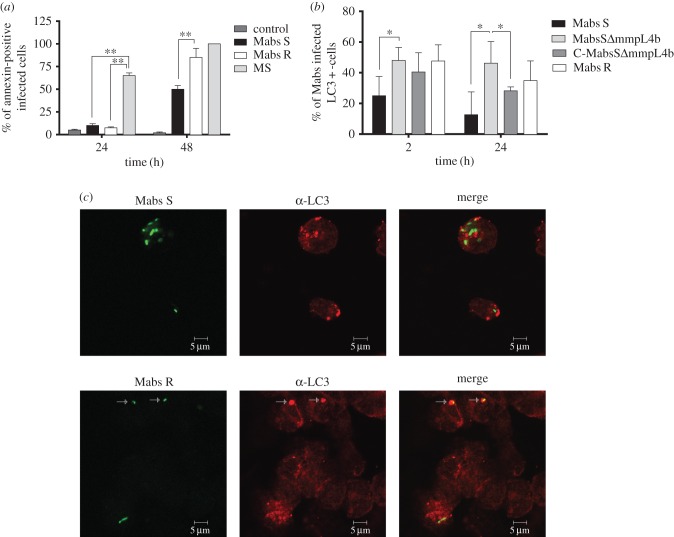
*Mycobacterium abscessus*-induced apoptosis and autophagy in wild-type Mϕ. (*a*) Analysis of apoptosis: THP-1 Mϕ were infected with *M. abscessus* S (Mabs S), *M. abscessus* R (Mabs R) or *M. smegmatis* (MS). The percentage of apoptotic cells was determined at 24 h p.i. using annexin-V-FITC conjugates (Abcam, USA) and propidium iodide to stain the dead cells. Fluorescent signals (mCherry from mycobacteria, FITC from the annexin-V and absence of propidium iodide) were analysed by flow cytometry. A significant increase in the percentage of apoptotic cells was associated with *M. smegmatis*-infected cells when compared with Mϕ infected with *M. abscessus* S or R. However, the R variant was significantly more pro-apoptotic than the S variant at 48 h. Error bars indicate the s.e.m. based on the results of two independent experiments (**p < 0.01). (*b*,*c*) Comparative behaviour of *M. abscessus* S and R variants towards autophagy. (*b*) Percentage of colocalization of *M. abscessus* S (Mabs S), S Δ*mmpL4b* mutant (MabsS ΔmmpL4b), Δ*mmpL4b* complemented (C-MabsS ΔmmpL4b) and R (Mabs R)-infected cells with LC3 at 2 and 24 h p.i. determined after immunofluorescence analyses. Error bars indicate the s.e.m. based on the results from four independent experiments (*p < 0.05). (*c*) Confocal immunofluorescence images of fixed THP-1 cells infected with Alexa-488-labelled *M. abscessus* variant S (Mabs S) or variant R (Mabs R) (green) (2 h p.i.) and immuno-stained for endogenous LC3 (red). Scale bars, 5 µm.

The phagosome–cytosol interplay may substantially impact on apoptosis and autophagy in the infected host phagocytes [49], both these cellular responses being efficient in controlling the intracellular growth of the RGM, *M. smegmatis* and *M. fortuitum* [24,50,51]. Here, we evaluated the extent of apoptosis following infection of THP-1 Mϕ with *M. abscessus* S, following annexin-V labelling. *M. smegmatis*, known as a potent apoptosis-inducing species [24,25], was also included as a positive control. Our results confirm the previously described pro-apoptotic activity of *M. smegmatis* [25] with nearly 80% of infected cells annexin-V-positive at 24 h p.i. (figure 6*a*). Compared to *M. smegmatis*, *M. abscessus* S- and R-infected Mϕ were only slightly apoptotic, with at most 10% of infected cells annexin-V-positive at 24 h (figure 6*a*). However, a significant difference in labelling was observed between the S and R variants at 48 h p.i. (figure 6*a*) with 50% and 90% of annexin-V-positive infected Mϕ, respectively. Overall, these results indicate that *M. abscessus* S is less pro-apoptotic than *M. abscessus* R, in agreement with previous observations in zebrafish [30].

Autophagy was next assessed after infection of THP-1 Mϕ with the Alexa488-labelled S variant and using anti-LC3-antibodies to specifically immunolabel autophagosome membranes. *M*. *abscessus* S did not co-localize with the LC3 marker, with 10% at 24 h p.i. of autophagosomes potentially containing the S variant (figure 6*b*,*c*). Comparatively, the R variant induced more autophagy than the S variant (figure 6*b*,*c*), as evidenced by the increased percentage of *M. abscessus*-LC3-positive infected cells (figure 6*b*) and confocal microscopy (figure 6*c*). In addition, the results obtained with the GPL-deficient Δ*mmpL4b* S mutant were comparable to those of *M. abscessus* R (figure 6*b*), confirming the importance of the surface-exposed GPL in the inhibition of autophagy of the S variant.

## 3. Discussion

The source and the mechanism responsible for pulmonary contamination of CF patients with *M. abscessus* remain unclear [52]. It appears, therefore, critical to understand how *M. abscessus* resists the Mϕ bactericidal activity, usually highly efficient against other RGM, such as *M. smegmatis* and *M*. *fortuitum* [24,25]. The S variant, capable of forming biofilm-like structures, is most likely to be the infecting form. GPL present at the surface of *M. abscessus* S have been shown to prevent TLR2 signalling in respiratory epithelial cells [53], possibly allowing lung colonization and subsequent survival in a silent and permissive niche, as shown for *M. tuberculosis* and *M. marinum* [20,45]. Herein, we confirmed that *M. abscessus* S survives in Mϕ, an observation consistent with previous studies [26–28], and demonstrated, for the first time, that replication of *M. abscessus* in Mϕ was not affected by functional CFTR defects. This provides evidence that such a modification is not responsible for the peculiar susceptibility of CF patients to *M*. *abscessus* infections, at least at the intracellular level. Noteworthily, Griffith *et al*. demonstrated that one third of the pulmonary infections due to *M. abscessus* can occur in the absence of a pre-existing lung pathology [2], emphasizing the intrinsic ability of *M. abscessus* to resist the bactericidal activity of Mϕ, a feature historically considered to be an exclusive attribute of SGM. In this context, Oberley-Deegan *et al*. proposed that *M. abscessus* could interfere with phagosome processing into phagolysosomes through the manipulation of host signal transduction pathways [27,54].

After phagocytosis by Mϕ, the S variant is found as a single bacterium in loner phagosomes whose membrane is closely apposed all around the outer surface of the mycobacterial cell wall. The S variant was also found to reside in slightly acidified non-mature phagosomes that are unable to fuse with lysosomes [42,43]. The retention of Rab5 at the membrane of *M. abscessus*-containing phagosomes in epithelial cells [54] is consistent with these findings. In addition, *M. abscessus* S was also found to be a poor apoptosis- and autophagy-inducing strain. These results were unexpected as all the RGM studied so far, particularly *M. smegmatis* and *M*. *fortuitum*, reside in phagolysosomes and are subsequently eliminated by the Mϕ. Moreover, RGM are known to induce the formation of autophagic vacuoles and cell apoptosis, both strategies considered important host cell defence mechanisms [55–57].

By contrast, the R form follows a rather different pathway. First, during the early infection stage, it presents a strong tendency to form chains or large clumps that usually remain in phagocytic cups instead of being internalized. As a result, cells contain less phagosomes but the latter contain generally multiple bacteria. It is well known that phagosomes containing several bacteria are systematically processed into phagolysosomes [17,23,41–44]. As expected, such phagosomes were rapidly acidified. Yet the intraphagosomal R variants did not appear to undergo degradation, at least during the first 24 h p.i., as assessed by EM observation of thin sections. The absence of degradation in phagolysosomes has already been observed for other mycobacteria [42]. Furthermore, the presence of the R variant within Mϕ induced the formation of autophagic vacuoles and cells became apoptotic. All these results point towards a typical RGM-like behaviour for the R variant [24,25]. It is noteworthy that the potent apoptosis-induced cell death activity may help the R variant to reach the extracellular environment ([30] and our study) where it can form cords. However, another unexpected result of this study was that the R variant, as opposed to other RGM, was able to replicate within Mϕ, an intriguing phenotype that may be due to the extensive clumping of the R variant within phagocytic cups or phagosomes. Moreover, re-invasion of extracellular bacteria that are released from the apoptotic Mϕ cannot be fully excluded.

The question that arises from this work is the following: why do the R and S variants follow such different phagosome trafficking pathways? Based on EM analyses of murine Mϕ infected with either *M. avium*, or *M. tuberculosis*, de Chastellier and colleagues showed evidence that, independent of the molecular mechanisms involved in the blocking of phagosome maturation of a mycobacterium-containing phagosome, the establishment and maintenance of a close apposition of the phagosome membrane with the entire mycobacterial surface all around represented a necessary requirement for prevention of phagosome maturation [39,42]. Interestingly, such a close interaction was observed in the case of S variant-containing phagosomes but not for those containing the R form. The establishment and maintenance of a long-lasting close apposition most probably involves the interaction between specific proteins and/or lipids of the phagosome membrane with specific components of the mycobacterial cell wall surface. Among these, ManLAM of *M. tuberculosis* is considered to be responsible for the prevention of phagosome maturation [58]. Recent studies also demonstrated that cyclopropane rings in α-mycolic acids are critical determinants for the phagosome maturation block [59]. The masking role of phenolic glycolipid (PGL), a major glycolipid found in SGM, including *M. marinum* and some *M. tuberculosis* clinical isolates, for recruiting permissive Mϕ has also been unravelled [60]. By analogy, GPL can be regarded as the PGL-matching glycolipid of atypical mycobacteria as it displays similar functional roles, such as limiting mycobacterial uptake [61] and masking the TLR2 ligands, thereby accounting for different inflammation-elicited responses towards S and R variants [36,62]. It could thus be argued that the S to R transition, involving loss of function mutations in the GPL biosynthetic or export machinery in *M. abscessus*, conditions the inflammatory response of the infected host.

The EM approaches used in this work provide compelling evidence on the role of GPL in the differential processing of phagosomes containing the S and R variants. It is well known that GPL is a major constituent of the mycobacterial cell wall ETZ [63,64]. While the ETZ was very thick in the case of the S variant, it was thinned out in the case of the R variant and also in the case of the Δ*mmpL4b* mutant that is unable to transport/assemble GPL at the bacterial surface. With the S variant being closely apposed to the phagosome membrane all around, and the R variant making contact with the phagosome membrane only at discrete sites, it is tempting to speculate that GPL is a major actor for establishing and maintaining the close apposition required for prevention of phagosome maturation. In fact, the *M. avium* complex GPL can delay phagosome–lysosome fusion following its ligation to the mannose receptor [65,66].

Interestingly, among *M. abscessus*, *M. smegmatis* and *M. chelonae*, all expressing cell surface GPL, as evidenced by high conservation of their respective *gpl* locus [67], only *M. abscessus* and *M. chelonae* have retained the ability to survive inside Mϕ [68]. *Mycobacterium smegmatis* mc^2^155 strain, which derives originally from the ATCC 607 strain [69], expresses less triglycosylated GPL than *M. abscessus* or *M*. *chelonae* [67]. That the level of GPL production can be enhanced in *M*. *smegmatis* mc^2^155 when overexpressing the whole *mbtH-mps1-mps2-gap* operon from *M*. *abscessus* [70] reflects quantitative differences in GPL production between these species, despite the presence of a similar *gpl* locus. Subtle differences at the cell surface in relation to GPL production may thus considerably interfere and modify the fate of mycobacteria within Mϕ.

Another important aspect concerns the possibility for *M. abscessus* to alter the phagosomal membrane and free itself from the phagosome in which it resides. A qualitative EM analysis of thin sections of at least 150 phagosomes showed that the membrane of S- but not R-containing phagosomes may present alterations. The membrane became loose and, in rare instances, showed breaks or partial lysis. Importantly, neighbouring organelles showed no signs of membrane alteration, thus ruling out eventual artefacts due to the processing of the EM samples. Unfortunately, we were neither able to quantify such events nor to conclusively determine whether the entire bacterium could be released into the cytosolic compartment.

The mycobacterial constituents and molecular mechanisms involved in the alteration or rupture of the *M. abscessus* S-containing phagosome membrane remain unknown. It has been reported that *M. abscessus* is not equipped with the ESX-1 apparatus, which mediates establishment of cytosol contact for slow-growing pathogenic mycobacteria such as *M. tuberculosis* [46,47,55,71,72], *M. marinum* [45] and *M. kansasii* [73]. Ongoing work dedicated to study a panel of defined transposon mutants [74] will hopefully allow us to identify the putative ESX-1-independent membrane-damaging constituents of the S strain, which are obviously mediated by an ESX-1-independent mechanism.

To summarize, it seems to be rather difficult to strictly compare the behaviours of S and R morphotypes. S and R variants can be regarded as two representatives of the same isolate, which coexist and/or evolve differently in response to host immunity, resulting in different fates for both the bacteria and the host. In conclusion, we provide compelling evidence that, at the cellular level, *M. abscessus* S imitates the phenotypic traits of pathogenic SGM and that the loss of cell wall associated lipids, namely GPL, can result in the acquisition of an RGM-like intracellular behaviour with a peculiar extracellular state characterized by a very high replication capacity [30]. CF patients are mainly infected with extracellular pathogens, such as *Pseudomonas* and *Staphylococcus*, with high growth rates in the bronchial lumen. By analogy, *M. abscessus* R can be regarded as the aggressive form in these patients. The capacity of *M. abscessus* to transition from an S intra-Mϕ-resistant form to an R extracellular form probably increases its capacity to adapt and survive in the changing environments. This is corroborated by recent comparative genomic studies indicating that *M. abscessus* evolves rapidly and should be monitored closely for the acquisition of more pathogenic traits. *Mycobacterium abscessus* genomes are very plastic, with many recently introduced insertion sequences such as prophages and novel genes, and have an open pan-genome [75,76], suggesting that they might continue to acquire new genetic material, for the adaptation to divergent environmental conditions.

## 4. Experimental procedures

### 4.1. Bacterial culture conditions

Isogenic pairs of S and R GFP- or mCherry-expressing *M. abscessus* CIP 104536T were used throughout this study. An *mmpL4b* KO (*ΔmmpL4b*) mutant displaying an R morphotype [34] and its complemented counterpart, which stably expresses MmpL4b under the control of the *hsp60* promoter, were also used. Mycobacteria were grown aerobically in Middlebrook 7H9 medium supplemented with 0.05% Tween 80 and 10% OADC (oleic acid, dextrose, catalase and bovine albumin) (Becton Dickinson, Le Pont-de-Claix, France) at 37°C. GFP-expressing strains were propagated in medium containing 500 µg ml^−1^ hygromycin B (Roche Diagnostics, Meylan, France). MCherry-expressing mycobacterial strains were grown in the presence of 250 µg ml^−1^ kanamycin (Sigma, USA).

### 4.2. Macrophage culture conditions

Animal experiments were performed according to institutional and national ethical guidelines (Agreement no 92-033-01). ΔF508 FVB mice, supplemented with movicol (Norgine, The Netherlands), and their wild-type FVB littermates [77,78] were from INRA (Jouy en Josas, France). The BMDM Mϕ, human monocyte- derived Mϕ (HMDM) and THP-1 Mϕ were prepared as previously described [31,79]. THP-1 Mϕ were cultured in RPMI 1640 supplemented with 10% FBS and differentiated for 24 h with 20 ng ml^−1^ PMA prior to infection.

### 4.3. Mϕ infections and intracellular growth measurement

Mycobacteria, grown aerobically at 37°C up to mid-log phase, were harvested by centrifugation and suspended in a phosphate buffer saline (PBS) solution (Sigma, USA). The bacterial clumps were disrupted by 20–30 passages through 26.5G insulin syringe and the bacterial suspension was then used to infect Mϕ (5 × 10^4^ to 10^5^) at an MOI between 1 and 10 mycobacteria per Mϕ in order to avoid rapid cell lysis, and incubated for 3 h at 37°C. After infection, cells were washed thoroughly with PBS (three to four times, except when explicitly stated differently) to eliminate extracellular bacteria and re-fed with complete medium containing amikacin at 250 µg ml^−1^ (except for EM experiments) for a further 1 h incubation at 37°C. This step was essential for killing the remaining extracellular mycobacteria, particularly the R variant that presents a sticky and clumpy phenotype (see Results). The medium containing amikacin was then discarded and cells were washed again three times. Infected cells were subsequently incubated in the presence of amikacin at 50 µg ml^−1^ at 37°C (except for EM experiments). CFU counts were performed at day 0 (or 4 h p.i. after the last wash), day 1, day 3 and day 5, by lysing the cells with cold distillated water, and plating 10-fold serial dilutions on LB plates (Sigma, USA). Colony enumeration was performed after 5–7 days of incubation at 37°C.

### 4.4. Analysis of phagosomal acidification

In total, 24-well plates of fully confluent monolayers of Mϕ were infected with FITC-labelled *M. abscessus* (expressing mCherry) at an MOI between 1 and 10, centrifuged at low speed for 90 s and incubated at 37°C for 15 min prior to fluorescence measurements. Fluorescent signals were then measured by sequentially exciting at 485 nm (FITC) and 544 nm (mCherry) using a Fluoroskan Ascent FL spectrofluorometer (Thermo Fisher Scientific, France). A standard pH curve was established to correlate the fluorescence signal with the pH as described [40].

### 4.5. Apoptotis and autophagy determination

The apoptosis assay was performed as described previously [80]. Mϕ were infected with *M*. *abscessus* and the percentage of apoptotic cells was determined using annexin-V–FITC conjugates (Abcam, USA) and propidium iodide staining of dead cells. Cells were infected with mCherry-expressing mycobacteria at an MOI of 10. For flow cytometry analysis, 10 000 events were collected for each condition and the percentages of annexin-V-positive mCherry-positive and propidium iodide-negative cells were determined, in order to count infected cells that became apoptotic.

For autophagy, mycobacteria were labelled with 50 µg ml^−1^ Alexa-488-succinimidyl ester in PBS for 45 min at room temperature (RT) and used to infect THP-1 Mϕ at an MOI of 1–10. Cells were spun down for 5 min at 800 rpm to synchronize phagocytosis, incubated for 30 min at 37°C, washed several times to remove extracellular bacteria and then incubated for 15 min, 2 h or 24 h at 37°C. Subsequently, cells were fixed with 2% paraformaldehyde (Delta microscopy) for 10 min, permeabilized with 0.1% Triton X100/PBS for 5 min and blocked with 4% BSA (Euromedex, France), 2% goat serum (Sigma, USA) in PBS for 1 h. Cells were incubated with rabbit anti-LC3 (MBL, France) overnight in blocking buffer, washed and then incubated for 3 h with secondary antibody coupled to Alexa-568. Observation was done using a Zeiss LSM 510 Inv confocal microscope and images were processed with ImageJ software. A total of 100 bacteria were counted per time point.

### 4.6. Processing for electron microscopy

Cells were fixed for 1 h at RT with 2.5% glutaraldehyde (Sigma, St Louis, MI, USA) in 0.1 M cacodylate buffer, pH 7.2, containing 0.1 M sucrose, 5 mM CaCl_2_ and 5 mM MgCl_2_. After two successive 15 min washes with the same buffer, cells were post-fixed for 1 h at RT with 1% osmium tetroxide (Electron Microscopy Sciences, distributed by Euromedex, Mundolsheim, France) in the same buffer devoid of sucrose. Cells were washed with buffer, scraped off the dishes with a rubber policeman, concentrated in 2% agarose in the same buffer, and treated for 1 h in 1% uranyl acetate in 30% methanol. Samples were dehydrated in a graded series of ethanol and embedded in Spurr resin. Thin sections were stained with 1% uranyl acetate in distilled water and then with lead citrate.

### 4.7. Fluorescence resonance energy transfer

We performed a modified assay that was previously used to investigate the breakdown of the endocytic vacuoles by Gram-negative bacteria using a chemical probe that is trapped within the host cytoplasm and detectable by fluorescence resonance energy transfer (FRET) measurements, as recently described [47]. Briefly, at successive stages of the time course measurements, a mix containing 50 µM CCF-4 substrate (Life Technologies, Saint Aubin, France) in EM medium (120 mM NaCl, 7 mM KCl, 1.8 mM CaCl2, 0.8 mM MgCl2, 5 mM glucose and 25 mM Hepes at pH 7.3) containing 2.5 µM probenicid was added to the infected THP-1 cells for 2 h at RT in the dark. Cells were then washed with PBS containing 2.5 µM probenicid before fixing with PFA 4% for 30 min at RT in the dark. Cells were washed directly before performing fluorescence imaging. Measurement of the ratio of 450 nm (blue)/535 nm (green) fluorescence allowed determining whether the bacteria established a phagosome–cytosol communication. Reading was performed by a fluorescence microscope with AutoPlay (Nikon) for the automatic acquisition of at least 50 cells per well and image analyses were performed using a dedicated algorithm using Metamorph software [47]. The experiment was repeated twice with similar results.

### 4.8. Statistical analyses

Fisher's exact test and the Student's *t*-test were used for all comparisons (GraphPad Prism version 6.0d; GraphPad Software, Inc). A *p*-value less than 0.05 was considered significant (n.s. = non-significant, *p < 0.05; **p < 0.01; ***p < 0.001; ****p < 0.0001).

## Supplementary Material

Sup. Figure 1: Preferential location of S variants of M. abscessus in loner phagosomes within BMDM.; Sup. Figure 2: In vitro growth of the S and R variants of M. abscessus.; Sup. Figure 3: Comparative intracellular growth of the S (A) and R (B) variants in wild type and ΔF508 murine Mф respectively.; Sup. Figure 4: M. abscessus S variant is able to damage the phagosome membrane of THP-1 cells as assessed by FRET.
