# Supplementary material for: The distinct fate of smooth and rough Mycobacterium abscessus variants inside macrophages
Source: Open Biol. 2016 Nov 30;6(11):160185. doi: 10.1098/rsob.160185 (PMC5133439; doi:10.1098/rsob.160185)
Supplement: Sup. Figure 1: Preferential location of S variants of M. abscessus in loner phagosomes within BMDM.; Sup. Figure 2: In vitro growth of the S and R variants of M. abscessus.; Sup. Figure 3: Comparative intracellular growth of the S (A) and R (B) variants in wild type and ΔF508 murine Mф respectively. [file rsob160185supp1.docx]

**
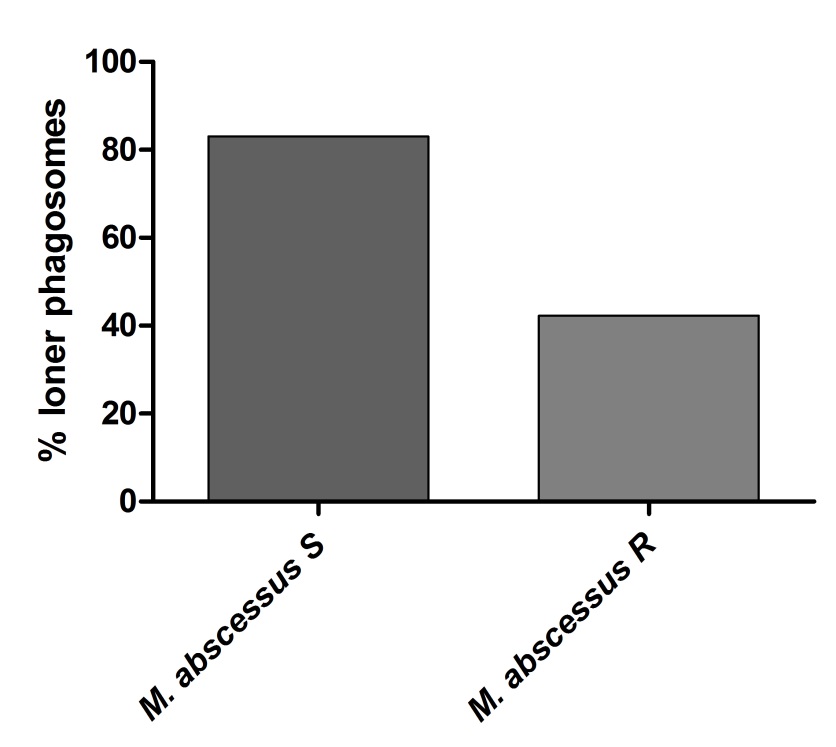
**

**Sup. Figure 1*:* Preferential location of S variants of *M. abscessus* in loner phagosomes within BMDM.**


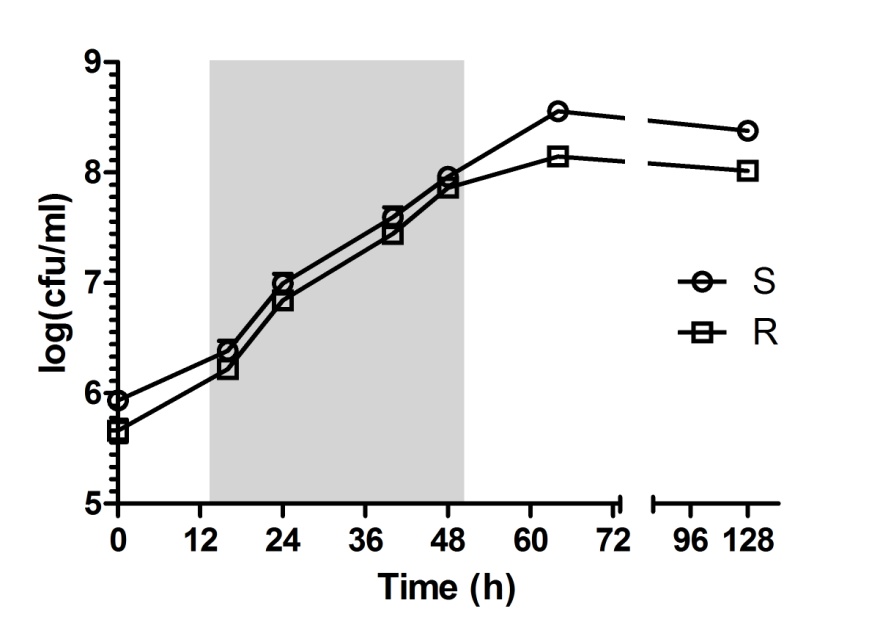


**Sup. Figure 2: *In vitro* growth of the S and R variants of *M. abscessus*.**

| A | B |
| --- | --- |
|   ns |  |

**Sup. Figure 3: Comparative intracellular growth of the S (A) and R (B) variants in wild type and ΔF508 murine Mф respectively.**


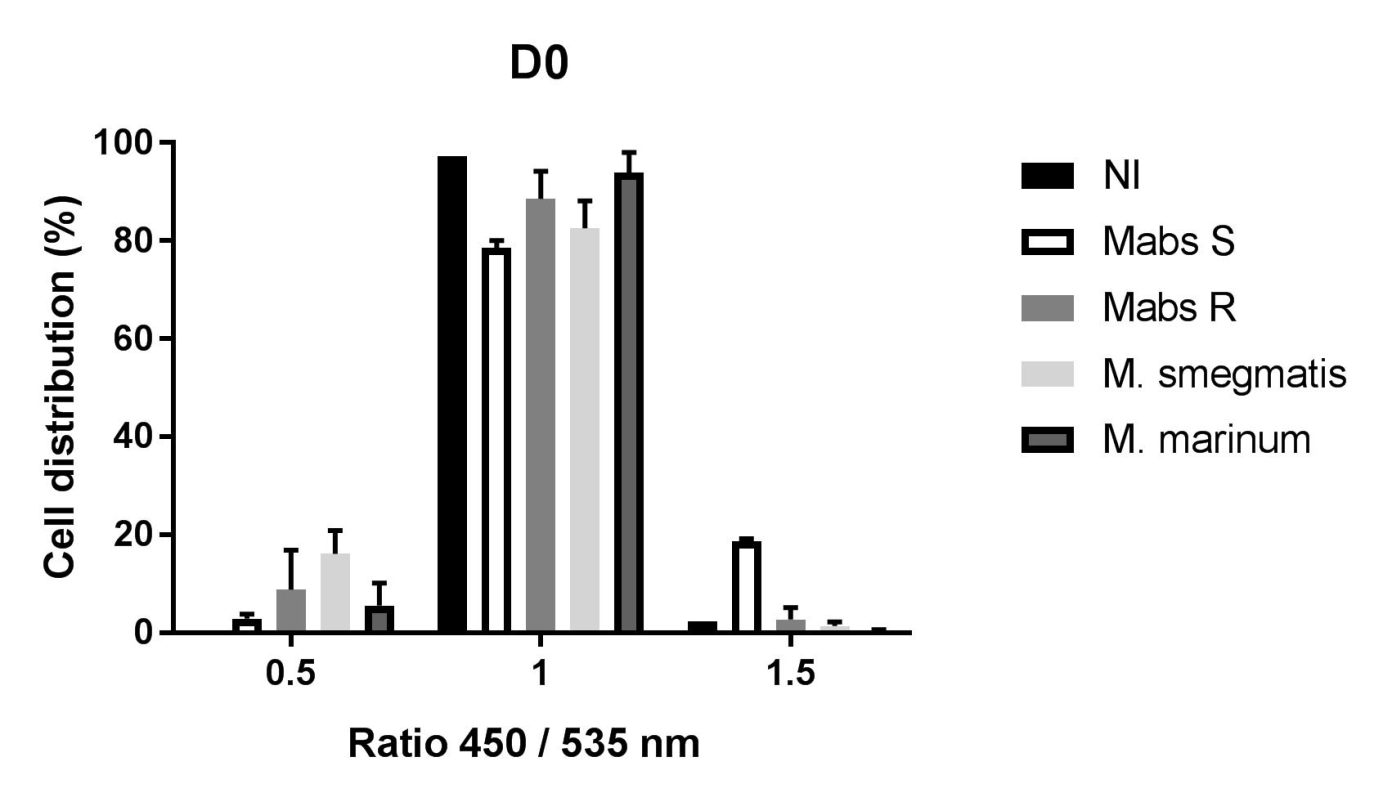


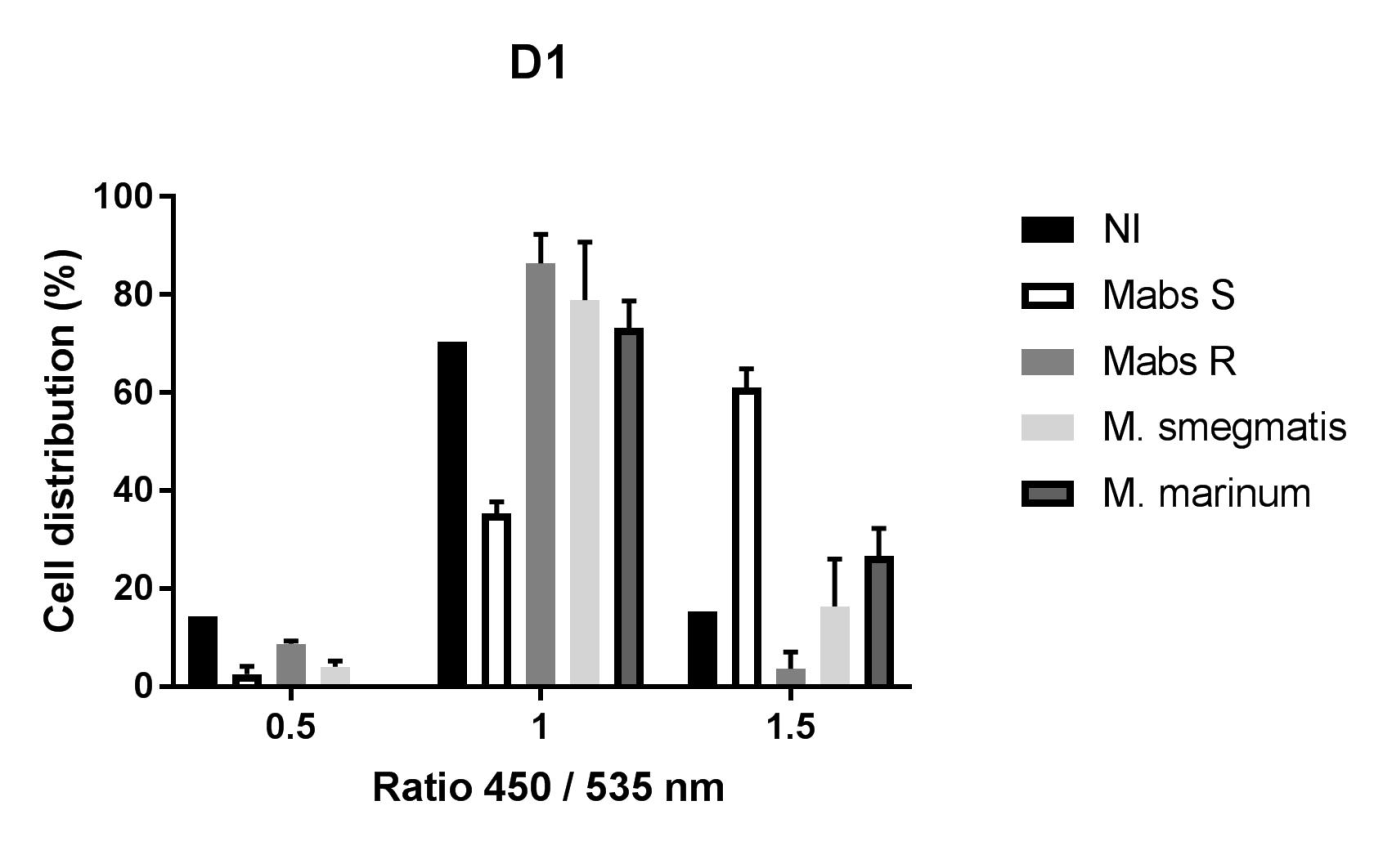


**Sup. Figure 4: *M. abscessus* S variant is able to damage the phagosome membrane of THP-1 cells as assessed by FRET.**
